# Clinical effectiveness of perioperative oxygen therapy strategies in children: a systematic review and meta-analysis of randomised controlled trials

**DOI:** 10.1016/j.bja.2025.12.003

**Published:** 2026-06-02

**Authors:** Adel Elfeky, Sara Tomassini, Rachel Court, Sara Bawa, Sophia Martin, Yen-Fu Chen, Amy Grove, Keith Couper, Joyce Yeung

**Affiliations:** 1 Warwick Evidence, University of Warwick, Coventry, UK; 2 Centre for Evidence and Implementation Science, School of Social Policy and Society, University of Birmingham, Birmingham, UK; 3 Warwick Clinical Trials Units, Warwick Medical School, University of Warwick, Coventry, UK; 4 Princess Alexandra Hospital NHS Foundation Trust, Harlow, UK; 5 Department of Critical Care Research, University Hospitals Birmingham NHS Foundation Trust, Birmingham, UK; 6 Department of Health and Welfare, University of Taipei, Taipei, Taiwan

**Keywords:** oxygen therapy, paediatric surgery, postoperative complications, surgical site infection, systematic review

## Abstract

**Background:**

Oxygen is routinely used in the perioperative period. However, its impact on clinical outcomes remains unclear. This systematic review aimed to assess the clinical effectiveness of oxygen strategies in paediatric patients undergoing surgical procedures.

**Methods:**

We searched MEDLINE, Embase, and Cochrane CENTRAL on May 1, 2025, for randomised controlled trials comparing perioperative oxygen strategies in children. Two reviewers independently identified eligible studies, extracted data, and assessed risk of bias and GRADE certainty in evidence. Meta-analyses were conducted with random effects models.

**Results:**

The review included 16 trials involving 1337 participants from 11 countries. Five trials compared intraoperative high fraction of inspired oxygen (Fio_2_, 60–80%) with low Fio_2_ (30–35%). In the postoperative period, four trials compared high-flow nasal oxygen (HFNO) with conventional oxygen therapy (COT), four trials investigated the effectiveness of noninvasive ventilation (NIV) compared with COT, and three trials compared HFNO with NIV. The evidence was very uncertain about the effect of high Fio_2_*vs* low Fio_2_ on surgical site infection (risk ratio [RR], 0.75; 95% confidence interval [CI], 0.33–1.73; risk difference [RD], −3%; 95% CI, −7.9% to +8.6%) and postoperative pulmonary complications (RR, 0.58; 95% CI, 0.24–1.42; RD, −5.4%; 95% CI, −9.8% to +5.4%). Postoperative HFNO use resulted in a large reduction in reintubation rate (RR, 0.34; 95% CI, 0.13–0.88; RD, −10.7%; 95% CI, −14.1% to −1.9%) compared with COT; however, GRADE certainty in evidence was low. The evidence was very uncertain about the effect of HFNO *vs* NIV use in the postoperative period on the incidence of reintubation (RR, 0.60; 95% CI, 0.26–1.37; RD, −5.2%; 95% CI, −9.7% to +4.8%) and pneumothorax (RR, 0.92; 95% CI, 0.11–7.76; RD, −0.5%, −5.9% to +45.1%)

**Conclusions:**

Current evidence does not support the routine use of any specific paediatric perioperative oxygen strategy. Further high-quality randomised trials are needed in this population.

**Systematic review protocol:**

PROSPERO (CRD42022331515).

**Editor’s key points**

• Oxygen is widely used in the perioperative period in children, but its effect on surgical site infections, pulmonary complications, and other outcomes remains uncertain; current guidelines lack paediatric-specific evidence.
• This systematic review shows very low-certainty evidence for all strategies. Postoperative high-flow nasal oxygenation might reduce reintubation *vs* conventional face mask oxygen, but data are limited and biased.
• No strategy should currently be routinely adopted. High-quality, adequately powered paediatric trials are urgently needed to guide practice.

Oxygen is routinely used in the perioperative setting.^1^ Perioperative oxygen therapy strategies encompass intraoperative administration of high inspired oxygen fractions and postoperative supplemental oxygen, aimed at mitigating hypoxaemia and maintaining an adequate safety margin against respiratory complications, which are recognised as a major contributor to severe adverse outcomes in paediatric surgical populations.^2^

Surgical site infections (SSIs) represent the most common healthcare-associated infection in the surgical paediatric population.^3^ Although less common than in adults (2.8% incidence *vs* 11% incidence), they represent a significant burden of disease and distress for patients and their caregivers.^4^^,^^5^ It is estimated that in ∼25% of cases, SSI is the leading cause of readmission after surgery with significant associated healthcare costs.^6^ Paediatric patients with congenital malformations, neonates, and those undergoing neurological or cardiac surgery represent high-risk groups for SSIs, which significantly increase postoperative morbidity and mortality.^7^^,^^8^

The World Health Organization (WHO) has published guidelines on the prevention of SSI in the adult population.^9^^,^^10^ It estimates that ∼50% of SSIs are fully preventable. WHO guidelines advocate the use of high fraction of inspired oxygen (Fio_2_) (>80%) in the perioperative period to prevent SSI.^9^^,^^10^ However, the WHO guidance published does not include children in its recommendations and there are no specific guidelines for oxygen therapy in the perioperative period for paediatric patients.^11^

Our overview of systematic reviews on the effectiveness of perioperative oxygen therapy identified no reviews in children.^12^ There is emerging research interest in investigating the benefits and risks of perioperative high Fio_2_ in children, but the guidelines on the use of supplemental perioperative oxygen remain inconsistent.^13^^,^^14^ There are similar uncertainty and variability in practice in relation to other perioperative oxygen strategies, such as noninvasive ventilation (NIV) strategies in the immediate postoperative period. A recent systematic review and meta-analysis examined the effectiveness of high-flow nasal oxygen compared with NIV in the postoperative period.^15^ A significant reduction in reintubation rates was reported (risk ratio [RR], 0.36; 95% confidence interval [95% CI], 0.25–0.53). The review was limited to paediatric patients who had undergone cardiac surgery and comprised of only one randomised trial and four observational studies.

Perioperative oxygen strategies potentially offer a relatively cheap and rapidly implementable way to reduce surgical morbidity and mortality. However, strategies must be proved to be clinically effective before they are recommended into routine practice. This systematic review aims to comprehensively evaluate the current evidence on the clinical effectiveness of perioperative oxygen therapy in children, including comparisons of high *vs* low Fio_2_, and the use of NIV strategies, such as continuous positive airway pressure (CPAP) and high-flow nasal oxygen (HFNO) *vs* conventional oxygen therapy (COT) in the postoperative period.

## Methods

We prospectively registered this review on PROSPERO (CRD42022331515) and have followed the Preferred Reporting Items for Systematic reviews and Meta-Analyses (PRISMA) guidelines.^16^ An *ad hoc* Advisory Group consisting of healthcare professionals involved in perioperative care and a patient was formed to provide clinical and methodological advice for this review and a companion overview of systematic reviews investigating the effectiveness of perioperative oxygen therapy.^12^

### Study selection criteria

Details of the inclusion and exclusion criteria are highlighted in Table 1.

**Table 1:** Overview inclusion and exclusion criteria. ∗Additional *post hoc* outcomes suggested by the advisory group.

Criteria	Inclusion	Exclusion
Population	Paediatric (<18 yr old) patients undergoing surgical procedures of any surgical specialty at any stage of the surgical pathway including the preoperative, intraoperative, and postoperative periods.	
Intervention	Perioperative oxygen therapy, defined as oxygenation strategy (e.g. high fraction of inspired oxygen, high-flow nasal oxygen, noninvasive ventilation) where the primary purpose of the intervention is to optimise oxygenation/oxygen delivery, with the aim of preventing hypoxaemia or reducing complications during the perioperative period.	We excluded randomised trials that primarily focused on intraoperative ventilation strategies (e.g. ventilatory rate, pressure, and volume settings), hyperbaric oxygen therapy, and extracorporeal life support. Studies that examined pre-oxygenation strategies for tracheal intubation were excluded.
Comparator	Any comparator or control.	
Outcomes	Primary outcomes: 1. Surgical site infection (SSI) within 30 days of follow-up after surgery—we followed definitions of the US Centers for Disease Control and Prevention (CDC) where possible. The CDC defines an SSI as an infection related to a surgical procedure that occurs near the surgical site within 30 days after surgery (or up to 90 days after surgery where an implant is involved).^17^ RCTs that have adopted other definitions were included and examined if they met other inclusion criteria, but differences in the outcome definitions were recorded and highlighted. 2. All-cause mortality within 30 days after surgery. Secondary outcomes: 1. Postoperative pulmonary complications: defined according to the most recent consensus^18^ as composite of respiratory diagnoses: (i) atelectasis detected on computed tomography or chest radiograph, (ii) pneumonia using US CDC criteria, (iii) acute respiratory distress syndrome (ARDS) using Berlin consensus definition, and (iv) pulmonary aspiration (clear clinical history AND radiological evidence). 2. Postoperative respiratory failure: including ARDS defined using Berlin consensus definition^19^ and need for mechanical ventilation. Definitions for the above outcomes are recommended by the StEP-COMPAC Group.^18^ We accepted similar outcomes defined differently in previous studies. Differences in the outcome definitions were recorded and highlighted. 3. Mortality up to the longest point of postoperative follow-up. 4. Length of hospital stay: the number of days from the day of surgery to hospital discharge or death. 5. ICU admission and number of days in ICU: unplanned admission to ICU within 14 days of surgery. 6. Postoperative nausea and vomiting.∗ 7. Pneumothorax.∗	
Study design	RCTs that examined the use of perioperative oxygen therapy in paediatric population.	

### Information sources and search strategy

Our information specialist searched MEDLINE, Embase, and the Cochrane Central Register of Controlled Trials (CENTRAL) from inception to May 1, 2025 (see Supplementary material). Searches were not limited by date or publishing language. We also reviewed the reference lists of relevant systematic reviews and included studies.

### Study selection

Titles and abstracts of records retrieved were screened by two reviewers independently and disagreement was resolved by discussion or, if needed, with the input of a third senior reviewer. Full-text articles considered potentially meeting inclusion criteria were assessed for inclusion by two reviewers independently and disagreements resolved as above. Non-English language articles were assessed by reviewers who can read the specific languages. We used Evidence for Policy and Practice Information (EPPI)-Reviewer 4 (EPPI-Centre, University College London, London, UK) software to manage records and data throughout the review.^20^

### Data extraction

Data were extracted to an Excel spreadsheet (Microsoft Corporation, Redmond, WA, USA) by one reviewer and then checked for accuracy by a second. Discrepancies were resolved through discussion. We extracted the following data from the included trials: country, number of patients randomised, age, type of surgery, stage of perioperative care, intervention, comparator, and clinical outcomes of interest.

### Risk of bias and certainty of evidence

Two reviewers independently assessed the risk of bias in the included RCTs using the Cochrane Risk of Bias 2 (RoB 2) tool.^21^ Disagreements were resolved through discussion or consultation with a third reviewer.

The GRADE (Grading of Recommendations Assessment, Development, and Evaluation) approach was used to assess the certainty of evidence for each outcome.^22^ GRADE assessment was undertaken by two reviewers independently using GRADEpro GDT software (Evidence Prime, Hamilton, Canada)^23^ and findings are reported using the informative statements guidance.^24^ When communicating an effect using these statements, we focused on the best estimate and on the certainty in that estimate which considers multiple factors. We communicated our findings using the terms ‘the evidence is very uncertain**’** to refer to very low certainty, ‘may**’** for low certainty, ‘probably**’** or ‘likely**’** for moderate level certainty, and simply the absence of qualification when referring to high certainty evidence on the effect of an intervention on a particular outcome (e.g. improves, reduces). Further details of the GRADE assessment are included in the Supplementary material.

### Data synthesis and analysis

Within each category, random effects meta-analysis was undertaken using STATA 17 software (StataCorp LLC, College Station, TX, USA) on groups of RCTs where interventions, comparators, and outcomes were deemed to be sufficiently homogeneous to allow statistical pooling of the results.^25^

When studies included more than two intervention arms, we excluded irrelevant groups or combined relevant groups as recommended in the Cochrane Handbook in order to avoid arbitrary decisions.^26^

Summary estimates with 95% CIs were obtained with the DerSimonian–Laird random effects model.^27^ We calculated RRs and risk difference (RD) with 95% CIs for SSI, postoperative pulmonary complications (PPCs), postoperative nausea and vomiting (PONV), reintubation rate, and incidence of atelectasis, and mean difference (MD) with 95% CIs for length of ICU stay. Studies where there were no events in both arms for a specific outcome were excluded from the meta-analysis as per Cochrane handbook recommendations.^28^^,^^29^

Where studies were sufficiently similar to be included in a meta-analysis, we assessed statistical heterogeneity by visually inspecting the forest plots, and calculating the *I*^2^ statistic.^25^ We planned, where feasible, to undertake the following subgroup analyses for the primary outcome. (i) Type of surgery: cardiac *vs* noncardiac. (ii) Patients at high risk of postoperative complications (as defined by investigators in each trial) *vs* those at low risk. (iii) Patient age: children *vs* neonates. (iv) Targeted use: preventive (preventing complications) *vs* therapeutic (treating hypoxaemia). (v) Studies with high risk of bias or those with some concerns *vs* studies with low risk.

We planned to use funnel plots and related methods to assess publication bias/small study effects if there was a sufficient number of studies (i.e. ≥10) included in the meta-analysis. Where outcome data from individual trials were not sufficiently homogeneous to be pooled in a meta-analysis, we used the synthesis without meta-analysis (SWiM) approach^30^ to provide a narrative description of results from clinically comparable trials.

## Results

**Fig 1:**
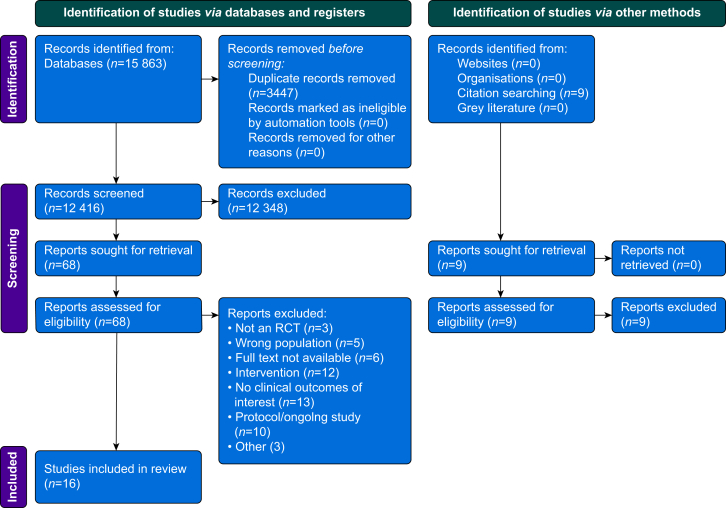
PRISMA flow diagram.

Our search identified 12 416 unique study records after duplicates were removed. After initial title and abstract screening, 68 full-text articles were assessed for eligibility. Sixteen randomised trials met our inclusion criteria and were included in the review. The PRISMA flow diagram is presented in Figure 1.

The included trials evaluated the effectiveness of oxygen therapy in paediatric patients undergoing various surgical procedures: cardiac surgery (*n*=7), abdominal (*n*=2), noncardiac surgery (*n*=1), orthopaedic (*n*=1), dental (*n*=1), tonsillectomy (*n*=1), subglottic balloon dilatation (*n*=1), any elective surgery lasting >2 h (*n*=1), and any surgery requiring general anaesthesia (*n*=1).

Five trials^13^^,^^31^, ^32^, ^33^, ^34^ examined the effect of intraoperative high Fio_2_ (60–80%) compared with low Fio_2_ (30–35%). Four trials^36^, ^37^, ^38^ assessed the effect HFNO compared with COT in the postoperative period. Four trials^39^, ^40^, ^41^, ^42^ investigated the effectiveness of postoperative NIV compared with COT, and three trials compared HFNO with NIV.^43^, ^44^, ^45^ See Table 2 for detailed characteristics of included RCTs. To reduce clinical heterogeneity, we grouped interventions into four categories based on their administration of oxygen in the perioperative care pathway and comparisons made: intraoperative high *vs* low Fio_2_, postoperative HFNO *vs* COT, postoperative NIV *vs* COT, and HFNO *vs* NIV.

**Table 2:** Characteristics of the included RCTs. CPAP, continuous positive airway pressure; COT, conventional oxygen therapy; HFNO, high-flow nasal oxygen; NIPPV, noninvasive positive pressure ventilation; PONV, postoperative nausea and vomiting; PPCs, postoperative pulmonary complications; SSI, surgical site infection.

Study	Country	No. of patients randomised	Age (mean)	Type of surgery	Perioperative care stage	Intervention *vs* control	Relevant outcomes
High *vs* low Fio_2_							
Behera (2021)^31^	India	132	8.5 yr	Abdominal and urological surgery	Intraoperative	80% Fio_2_*vs* 30% Fio_2_	PONV, SSI, PPCs
Donaldson (2005)^32^	Australia	95	6.2 yr	Dental	Intraoperative	80% Fio_2_*vs* 30% Fio_2_	PONV
de la Grandville (2019)^13^	Switzerland	58	12.5 yr	Non-abdominal, non-thoracic surgery (mostly orthopaedic)	Intraoperative	80% Fio_2_*vs* 35% Fio_2_	Respiratory adverse events, PONV, SSI
Izadi (2016)^33^	Iran	103	7.9 yr	Tonsillectomy	Intraoperative	80% Fio_2_*vs* 30% Fio_2_	PONV
Song (2019)^34^	South Korea	86	2.2 yr	Noncardiac surgery	Intraoperative	60% Fio_2_*vs* 30% Fio_2_	Incidence of atelectasis, PPCs
HFNO *vs* COT							
Enayati (2021)^35^	Iran	105	9.1 months	Cardiac surgery	Postoperative	HFNO *vs* COT	Respiratory failure, reintubation, PPCs, length of ICU stay
Kumar (2022)^36^	India	127	45 months	Cardiac surgery	Postoperative	HFNO *vs* COT	Reintubation, length of ICU stay
Lee (2021)^37^	South Korea	80	10.3 months	Elective surgery lasting >2 h	Postoperative	HFNO *vs* COT	Atelectasis, pneumonia, respiratory adverse events, length of hospital stay
Testa (2014)^38^	Italy	94	3.36 months	Cardiac surgery	Postoperative	HFNO *vs* COT	Atelectasis, escalation of respiratory support, reintubation
NIV *vs* COT							
Abdel-Ghaffar (2019)^39^	Egypt	60	4 yr	Laparoscopic abdominal surgery	Postoperative	CPAP *vs* COT	PONV
Acosta (2021)^40^	Argentina	42	4 yr	Any surgery requiring general anaesthesia	Intra and postoperative	CPAP *vs* COT	Atelectasis
Silva (2016)^41^	Brazil	62	11.5 yr	Cardiac surgery	Postoperative	CPAP *vs* COT	Length of hospital stay, length of ICU stay
Tuncel (2025)^42^	Turkey	81	2.28 yr	Subglottic balloon dilatation	Postoperative	CPAP *vs* COT	ICU admission, intubation
HFNO *vs* NIV							
Goel (2024)^45^	India	40	16.35 months	Cardiac surgery	Postoperative	HFNO *vs* NIPPV	Reintubation, atelectasis, pneumothorax
Wu (2021)^43^	China	80	2.25 months	Cardiac surgery	Postoperative	HFNO *vs* CPAP	Reintubation, respiratory failure, mortality, length of hospital stay
Wu (2022)^44^	China	92	1.45 months	Cardiac surgery	Postoperative	HFNO *vs* NIPPV	Reintubation

### Risk of bias in included studies

**Fig 2:**
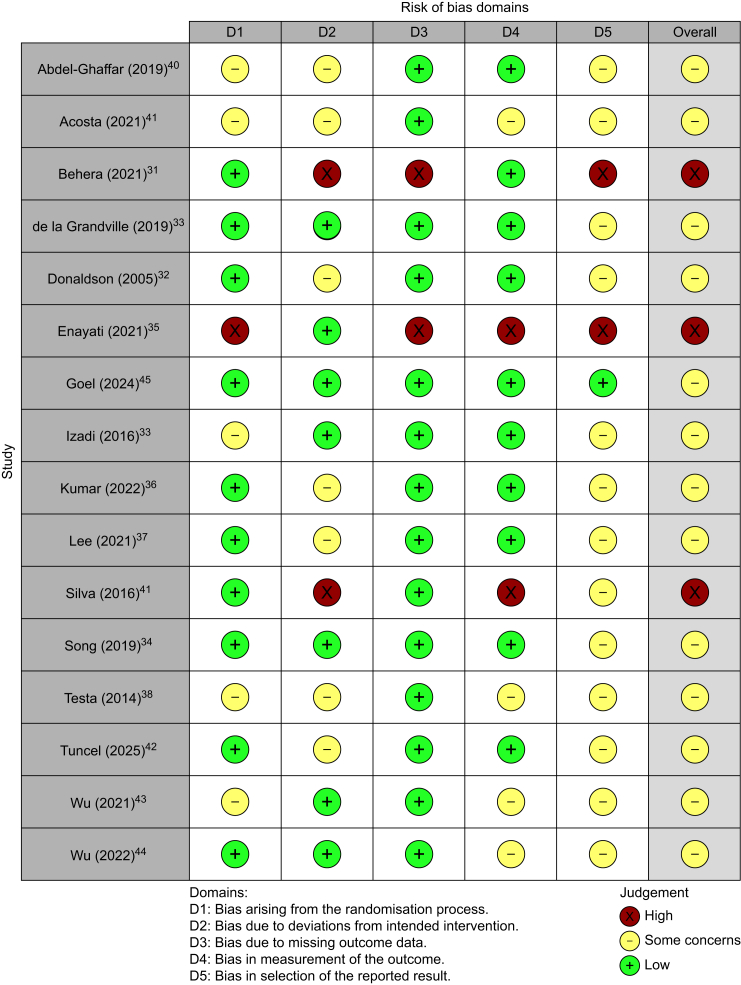
Risk of bias summary: review authors**’** judgements about each risk of bias item for each included trial.

We deemed three trials to be at high risk of bias^31^^,^^35^^,^^41^ and classified 13 trials^13^^,^^32^, ^33^, ^34^^,^^36^, ^37^, ^38^, ^39^, ^40^^,^^42^, ^43^, ^44^, ^45^ as having some concerns for risk of bias. This was largely driven by concerns arising from lack of published information to confirm whether reported results in these trials were selected and analysed in accordance with a pre-specified analysis plan. Our risk of bias assessment is presented in Figure 2.

### Meta-analyses

Meta-analysis was conducted where possible for three comparison categories (high *vs* low Fio_2_, HFNO *vs* COT, and HFNO *vs* NIV) where multiple studies were found with comparable treatment arms. A detailed summary of findings for all outcomes across the four intervention categories is presented in Table 3. Forest plots and GRADE evidence profiles are provided in the Supplementary material. Overall, certainty in the evidence was very low for most of the outcomes of interest. This is largely driven by imprecision in estimates because of limited sample size of trials reporting on each outcome.

**Table 3:** Summary of findings across all intervention categories. Bold type indicates statistically significant difference between groups. COT, conventional oxygen therapy; GRADE, Grading of Recommendations Assessment, Development, and Evaluation; HFNO, high-flow nasal oxygen; MD, mean difference; NIV, noninvasive ventilation; PONV, postoperative nausea and vomiting; PPC, postoperative pulmonary complication; RD, risk difference; RR, risk ratio; SSI, surgical site infection.

Outcomes	Study ID	Number of studies (total participants)	Relative effect estimates (95% confidence interval)	Absolute effect estimates (95% confidence interval)	GRADE certainty of evidence
High *vs* low Fio_2_					
SSI	Behera (2021),^31^ de la Grandville (2019)^33^	2 (186)	RR, 0.75 (0.33–1.73)	RD, −0.03 (−0.08 to 0.09)	Very low certainty
Mortality		0	-	-	-
PPC	Behera (2021),^31^ de la Grandville (2019)^33^	2 (186)	RR, 0.58 (0.24–1.42)	RD, −0.05 (−0.1 to 0.05)	Very low certainty
Postoperative respiratory failure		0	-	-	-
Reintubation		0	-	-	-
Length of hospital or ICU stay		0	-	-	-
Unplanned ICU admission		0	-	-	-
PONV	Behera (2021),^31^ Donaldson (2005),^32^ de la Grandville (2019),^13^ Izadi (2016)^33^	4 (383)	RR, 0.82 (0.57–1.17)	RD, −0.043 (−0.10 to 0.04)	Very low certainty
Atelectasis	Song (2019)^34^	1 (86)	RR, 1.33 (0.72–2.47)	RD, 0.092 (−0.08 to 0.41)	Very low certainty
HFNO *vs* COT					
SSI		0	-	-	-
Mortality		0	-	-	-
PPC		0	-	-	-
Postoperative respiratory failure		0	-	-	-
Reintubation	Enayati (2021),^35^ Kumar (2022),^36^ Testa (2014)^38^	3 (302)	RR, 0.34 (0.13–0.88)	RD, −0.11 (−0.14 to −0.02)	Low certainty
Length of ICU stay (days)	Enayati (2021),^35^ Kumar (2022),^36^ Testa (2014)^38^	3 (302)	-	MD, −0.05 (−1.1 to 0.15)	Very low certainty
Unplanned ICU admission		0	-	-	-
PONV		0	-	-	-
Atelectasis	Lee (2021),^37^ Testa (2014)^38^	2 (167)	RR, 0.35 (0.03–3.57)	RD, −0.08 (−0.13 to 0.34)	Very low certainty
NIV *vs* COT					
SSI		0	-	-	-
Mortality		0	-	-	-
PPC		0	-	-	-
Postoperative respiratory failure		0	-	-	-
Intubation	Tuncel (2025)^42^	1 (84)	RR, 1.46 (0.62–3.47)	RD, 0.079 (−0.06 to 0.42)	Very low certainty
Length of hospital stay (days)	Silva (2016)^41^	1 (50)	-	MD, −1.2 (−3.6 to 1.2)	Very low certainty
Length of ICU stay (days)	Silva (2016)^41^	1 (50)	-	MD, 0.5 (−0.1 to 1.1)	Very low certainty
Unplanned ICU admission	Tuncel (2025)^42^	1 (84)	RR, 0.85 (0.42–1.75)	RD, −0.04 (−0.17 to 0.22)	Very low certainty
Postoperative vomiting	Abdel-Ghaffar (2019)^39^	1 (60)	RR, 0.82 (0.44–1.53)	RD, −0.1 (−0.30 to 0.29)	Very low certainty
Atelectasis	Acosta (2021)^40^	1 (42)	RR, 0.32 (0.07–1.39)	RD, −0.20 (−0.28 to 0.12)	Very low certainty
HFNO *vs* NIV					
SSI		0	-	-	-
Mortality (hospital)	Wu (2021)^43^	1 (80)	RR, 0.33 (0.01–7.95)	RD, −0.02 (−0.02 to 0.17)	Very low certainty
PPC—pneumothorax	Goel (2024),^45^ Wu (2021)^43^	2 (120)	RR, 0.92 (0.11–7.76)	RD, −0.005 (−0.06 to 0.45)	Very low certainty
Postoperative respiratory failure	Wu (2021)^43^	1 (80)	RR, 0.33 (0.10–1.14)	RD, −0.15 (−0.20 to 0.03)	Very low certainty
Reintubation	Goel (2024),^45^ Wu (2021),^43^ Wu (2022)^44^	3 (212)	RR, 0.60 (0.26–1.37)	RD, −0.05 (−0.1 to 0.05)	Very low certainty
Length of hospital or ICU stay		0	-	-	-
Unplanned ICU admission		0	-	-	-
PONV		0	-	-	-
Atelectasis		0	-	-	-

### Intraoperative high *vs* low fraction of inspired oxygen

No trials reported on 30-day mortality or mortality up to the longest point of postoperative follow-up for this comparison.

**Fig 3:**
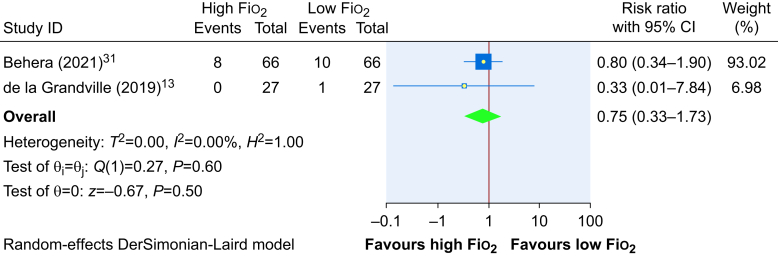
Effect of high Fio_2_ on SSI when compared with low Fio_2_. Fio_2_, fraction of inspired oxygen; 95% CI, 95% confidence interval; SSI, surgical site infection.

The meta-analysis on the primary outcome of SSI included only two trials,^13^^,^^31^ representing 186 patients. The evidence is very uncertain about the effect of intraoperative high Fio_2_ on SSI (RR, 0.75; 95% CI, 0.33–1.73; RD, −3%; 95% CI, −7.9% lower to +8.6%). The forest plot is presented in Figure 3 and GRADE evidence profile is provided in the Supplementary material.

Two trials^13^^,^^31^ including 186 patients reported on PPCs. Compared with low Fio_2_, the evidence is very uncertain about the effect of high Fio_2_ on PPCs (RR, 0.58; 95% CI, 0.24–1.42; RD, −5.4%; 95% CI, −9.8% to +5.4%) (Supplementary Fig. 1). Four trials^13^^,^^31^, ^32^, ^33^ including 383 patients reported on PONV. Compared with low Fio_2_, the evidence is very uncertain about the effect of high Fio_2_ on the incidence of PONV (RR, 0.82; 95% CI, 0.57–1.17; RD, −4.3%; 95% CI, −10.4% to +4.1%) (Supplementary Fig. 2). Only one trial reported on the effect of high Fio_2_ (60%) on the development of atelectasis compared with low Fio_2_ (30%) in mechanically ventilated children.^34^ The incidence of significant atelectasis on the postoperative lung ultrasound was similar between the two groups (RR, 1.33; 95% CI, 0.72–2.47; RD, −9.2%; −7.8% to +41%).

### Postoperative high-flow nasal oxygen *vs* conventional oxygen therapy

No trials reported on 30-day mortality or mortality up to the longest point of postoperative follow-up for this comparison.

Three trials^35^^,^^36^^,^^38^ including 302 patients reported on reintubation rate. Compared with COT, HFNO use in the postoperative period may result in a large reduction in reintubation rate (RR, 0.34; 95% CI, 0.13–0.88; RD, −10.7%; 95% CI, −14.1% to −1.9%). GRADE certainty in evidence was low (Supplementary Fig. 3).

Two trials^37^^,^^38^ including 167 patients reported on atelectasis. Compared with COT, the evidence is very uncertain about the effect of HFNO on the incidence of atelectasis (RR, 0.35; 95% CI, 0.03–3.57; RD, −8.5%; 95% CI, −12.7% to +33.7%) (Supplementary Fig. 4).

Three trials^35^^,^^36^^,^^38^ including 302 patients reported on length of ICU stay. Compared with COT, the evidence is very uncertain about the effect of HFNO on the length of ICU stay (MD, −0.46 days; 95% CI, −1.06 to +0.15) (Supplementary Fig. 5).

### Postoperative noninvasive ventilation *vs* conventional oxygen therapy

Meta-analysis was not possible on any clinical outcome of interest. None of the included trials reported on 30-day mortality or mortality up to the longest point of postoperative follow-up for this comparison.

One randomised trial examined the effect of prophylactic CPAP application on airway complications in 84 paediatric patients with subglottic stenosis undergoing balloon dilatation.^42^ No significant difference was found between the CPAP and non-CPAP groups in the incidence of unplanned ICU admission (RR, 0.85; 95% CI, 0.42–1.75; RD, −4.4%; 95% CI, −17% to +22%) and intubation rate (RR, 1.46; 95% CI, 0.62–3.47; RD, +7.9%; 95% CI, −6.5% to +42.2%).

Only one trial reported on the effect of CPAP in preventing postoperative atelectasis in children under general anaesthesia.^40^ Compared with standard care, CPAP did not significantly reduce the incidence of atelectasis (RR, 0.32; 95% CI, 0.07–1.39; RD, −20.4%; 95% CI, −27.9% to +11.7%).

In a randomised trial with 50 children who had undergone cardiac surgery,^41^ CPAP did not reduce the number of days spent in the hospital (MD, −1.2 days; 95% CI, −3.6 to +1.2) and ICU (MD, 0.5 days; 95% CI, −0.1 to +1.1) compared with standard care.

In a randomised trial comparing post-extubation CPAP with standard care in 60 patients after paediatric laparoscopic surgery,^39^ no significant difference in postoperative vomiting was found between the groups (RR, 0.82; 95% CI, 0.44–1.53; RD, −9.9%; 95% CI, −30.8% to +29.2%).

### Postoperative high-flow nasal oxygen *vs* noninvasive ventilation

Three trials^43^, ^44^, ^45^ including 212 patients reported on reintubation rate. The evidence is very uncertain about the effect of postoperative HFNO on reintubation compared with NIV (RR, 0.60; 95% CI, 0.26–1.37; RD, −5.2%; 95% CI, −9.7% to +4.8%) (Supplementary Fig. 6).

The evidence from two trials^43^^,^^45^ including 120 patients is very uncertain about the effect of HFNO on the incidence of pneumothorax compared with NIV (RR, 0.92; 95% CI, 0.11–7.76; RD, −0.5%; 95% CI, −5.9% to +45.1%) (Supplementary Fig. 7).

Only one trial reported on post-extubation respiratory failure and hospital mortality. The study evaluated the effects of HFNO compared with nasal CPAP in 80 infants after congenital heart surgery.^43^ There was no significant difference between the two groups in the incidence of respiratory failure (RR, 0.33; 95% CI, 0.1–1.14; RD, −15.1%; 95% CI, −20.3% to +3.1%) and hospital mortality (RR, 0.33; 95% CI, 0.01–7.95; RD, −1.7%; 95% CI, −2.5% to +17.4%).

### Subgroup analyses

We could not explore potential subgroup differences by type of surgery, sex, patient age (children *vs* neonates), and targeted use of the intervention (preventive *vs* therapeutic) owing to limited stratified analyses in primary studies.

## Discussion

### Summary of main results and certainty of evidence

This systematic review, which included meta-analyses of data from 16 trials across four comparison categories, is the most comprehensive synthesis of evidence on the clinical effectiveness of perioperative oxygen therapy in paediatric patients to date. We found that evidence is very uncertain about the effect of intraoperative high Fio_2_ on SSI and PPCs compared with low Fio_2_. For all clinical outcomes including reintubation rate and incidence of atelectasis, the evidence is very uncertain about the effect of postoperative HFNO or NIV compared with COT or compared against each other. The only statistically significant finding of clinical importance was a substantial reduction in reintubation for HFNO compared with COT (Table 3, Supplementary Fig. 3). However, only three relatively small trials contributed data towards this pooled estimate, with the largest trial that drove the result being judged to be of high risk of bias in multiple domains. The finding, therefore, requires further confirmation.

The lack of high-quality evidence means that paediatric clinical practice guidelines are frequently based on expert consensus and attempts to extrapolate indirect evidence from adults.^46^^,^^47^ Although evidence on the effectiveness of perioperative oxygen strategies in adults is abundant, its quality remains low and further large-scale randomised trials are required to reduce uncertainty.^12^ This underscores the extreme lack of robust evidence to guide clinical decision-making in paediatric anaesthesia. As a result, guidelines on the use of supplemental perioperative oxygen remain inconsistent, and there is little clarity on the approaches anaesthetists are adopting in routine clinical practice. An international survey among paediatric anaesthesiologists in Europe, USA, Australia, and New Zealand was conducted to assess their daily practices regarding oxygen use during noncardiac surgery.^48^ The survey revealed that oxygen administration practices during paediatric anaesthesia are minimally regulated, reflecting a lack of standardisation across clinical settings; 87% of respondents reported to titrate Fio_2_ intraoperatively, mainly based on pulse oximetry values. The reported median standard percentage of intraoperative oxygen administration was 35% (interquartile range [IQR], 30–40%). This pragmatic approach reflects the current uncertainty surrounding the balance of potential benefits and harms associated with supplemental oxygen in paediatric anaesthesia. Current practice remains largely unstandardised because of insufficient evidence, highlighting an urgent need for well-designed paediatric trials. To strengthen clinical guidance, future studies should address heterogeneity in age groups, sex, underlying conditions, and perioperative risk profiles, enabling robust, generalisable evidence to guide paediatric perioperative oxygen strategies.

### Strengths and limitations of this review

Despite the strengths, such as the comprehensiveness of the search strategies and the use of GRADE approach to assess the certainty in evidence, several limitations should be considered. First, our meta-analyses were limited by the small number of studies for all outcomes of interest; therefore, the findings should be interpreted as exploratory. Second, because of limited stratified analyses in primary studies, we could not explore potential subgroup differences by type of surgery, patient age (children *vs* neonates), and targeted use of the intervention (preventive *vs* therapeutic). Most of the included trials did not report sex-disaggregated data, and we were unable to incorporate sex into our analyses or models. Thirdly, the review is made up of studies with small sample sizes and poor methodological quality. Finally, we have incorporated evidence on both high inspired oxygen and postoperative ventilation modalities as independent strategies within the review. We acknowledge that these interventions can exert opposing physiological effects; for example, high Fio_2_ can increase the risk of atelectasis, whereas HFNO might reduce it. However, because of limitations in the available data, we were unable to explore potential interactions between these strategies in our analysis.

### Conclusions

Current evidence is insufficient to support the routine use of perioperative oxygenation strategies in children. Future studies should aim to close this knowledge gap through well-designed, adequately powered randomised controlled trials.

## Authors’ contributions

Led the review, maintained day-to-day running of the project, was involved in all stages of the review process: AE

Drafted the manuscript: AE, YFC, AG, KC, JY

Revised the manuscript: AE, YFC, AG, KC, JY, SB, SM

Review design and study conduct: YFC, AG, KC, JY

Led the database search, designed and ran the searches for the systematic overview, helped manage the references and referencing: RC

Title/abstract screening, full-text screening, data extraction, quality assessment, writing the manuscript: ST

Screening and data extraction: SB, SM

Attests that all listed authors meet authorship criteria and that no others meeting the criteria have been omitted: JY

## Declarations of interest

YFC is a member of the NIHR Evidence Synthesis Programme Prioritisation and Advisory Group. AG is a member of the Member of the NIHR HTA Commissioning Committee and the NIHR DSE Fellowship Funding Committee. KC is a member of NIHR HTA hospital-based prioritisation committee and NIHR RfPB West Midlands funding committee. JY is a member of NIHR HTA General Committee. The other authors declare that they have no conflicts of interest.
