# Supplementary material for: Clinical effectiveness of perioperative oxygen therapy strategies in children: a systematic review and meta-analysis of randomised controlled trials
Source: Br J Anaesth. 2026 Jun 2;136(4):1307–18. doi: 10.1016/j.bja.2025.12.003 (PMC13014514; doi:10.1016/j.bja.2025.12.003)
Supplement: Multimedia component 1 [file mmc1.docx]

**Clinical effectiveness of perioperative oxygen therapy in paediatric population: A systematic review and meta-analysis of randomised controlled trials**

**Supplementary material**

Table of Contents

[Search strategy 1](#_Toc210403082)

[Further details of GRADE assessment 9](#_Toc210403083)

[Figure S1: Effect of high FiO_2_ on PPCs when compared to low FiO_2_ 10](#_Toc210403084)

[Figure S2: Effect of high FiO_2_ on PONV when compared to low FiO_2_ 10](#_Toc210403085)

[Figure S3: Effect of HFNO on reintubation when compared to COT 11](#_Toc210403086)

[Figure S4: Effect of HFNO on the incidence of atelectasis when compared to COT 11](#_Toc210403087)

[Figure S5: Effect of HFNO on length of ICU stay when compared to COT 12](#_Toc210403088)

[Figure S6: Effect of HFNO on reintubation when compared to NIV 12](#_Toc210403089)

[Figure S7: Effect of HFNO on the incidence of pneumothorax when compared to NIV 12](#_Toc210403090)

[GRADE evidence profile-High FiO_2_ compared to low FiO_2_ 14](#_Toc210403091)

[GRADE evidence profile- HFNO compared to COT 16](#_Toc210403092)

[GRADE evidence profile- NIV compared to COT 18](#_Toc210403093)

[GRADE evidence profile- HFNO compared to NIV 20](#_Toc210403094)

# Search strategy

| **Source(s) and date coverage** | **Date searched (date updated)** | **Description of search** | **Number retrieved + update** | **Notes** |
| --- | --- | --- | --- | --- |
| MEDLINE (Ovid)  MEDLINE(R) ALL <1946 to August 22, 2022>  Update 1: Ovid MEDLINE(R) ALL <1946 to July 18, 2023>  Update 2: Ovid MEDLINE(R) ALL <1946 to April 30, 2025> | 23/08/2022  (19/07/2023 and 01/05/2025) | Children, perioperative oxygen and RCTs | 3362 + 157 + 344 | Used the ‘Children (broad)’ expert search for MEDLINE in Ovid (which is based on the ‘new CCG’ filter designed for Pubmed). The perioperative oxygen subject search was developed in CENTRAL for our perioperative oxygen NIHR project. The Cochrane sensitivity and precision maximising RCT filter is used and supplemented with a few additional RCT terms to increase sensitivity.  The update searches used the 2023 revision of Cochrane sensitivity and precision maximising RCT filter and database entry date to limit it to records added to the database after the original search. “2023 update: The MeSH 'Randomized controlled trial' now has more specific headings, so the Ovid version of the filter has been edited to explode that subject heading” |
| Embase (Ovid)  Embase Classic+Embase <1947 to 2022 August 22>  Update 1: Embase Classic+Embase 1947 to 2023 July 18  Update 2: Embase 1974 to 2025 May 01 | 23/08/2022  (19/07/2023 and 02/05/2025) | Children, perioperative oxygen and RCTs | 5065 + 877 + 1757 | Used the ‘Children (broad)’ expert search for Embase in Ovid. The perioperative oxygen subject search is translated from the search developed in CENTRAL for our perioperative oxygen NIHR project. The Cochrane RCT filter is used <https://www.cochranelibrary.com/central/central-creation>.  The update searches used the 2023 revision of the Cochrane RCT filter and database entry date to limit it to records added to the database after the original search. |
| CENTRAL (Cochrane Library via Wiley) | 23/08/2022  (19/07/2023 and 02/05/2025) | Children and perioperative oxygen | 3604 + 224 + 483 | Translation of MEDLINE ‘Children (broad)’ filter (which is based on the ‘new CCG’ filter designed for Pubmed). Did not apply an RCT filter in CENTRAL.  Update searches used the Cochrane Library publication date to limit it to records added to the database after the original search. |

**Totals for PRISMA flow diagram(s):**

Total from databases: 12,021

Total after duplicates removed: 9,273

1^st^ Update Search: Total from databases: 1,258

1^st^ Update Search: Total after duplicates removed: 1,112

1^st^ Update Search: Total after duplicates with original search removed: 1,030

2^nd^ Update Search: Total from databases: 2,584

2^nd^ Update Search: Total after duplicates removed: 2,242

2^nd^ Update Search: Total after duplicates with original and update search removed: 2,113

If combining original and update searches into one PRISMA

Total from databases: 12,021 + 1,258 + 2,584 = 15,863

Total after duplicates removed: 9,273 + 1,030 + 2,113 = 12,416

**Medline (Ovid)**

Note: details of the 2023 and 2025 update searches are at the end of the search string.

Date searched: 23/08/2022

Ovid MEDLINE(R) ALL <1946 to August 22, 2022>

1 exp adolescent/ or exp child/ or exp infant/ or (infant disease* or childhood disease*).ti,ab,kf. or (adolescen* or babies or baby or boy? or boyfriend or boyhood or girlfriend or girlhood or child* or girl? or infan* or juvenil* or kid? or minors or minors* or neonat* or neo-nat* or newborn* or new-born* or paediatric* or peadiatric* or pediatric* or perinat* or preschool* or puber* or pubescen* or school* or teen* or toddler? or underage? or under-age? or youth*).ti,ab,kf. or (pediatric* or paediatric* or infan* or child* or adolescen* or young).jn,jw. or (pediatric* or paediatric* or infan* or child* or adolescen* or young).in. 5490848

2 exp Specialties, Surgical/ 214932

3 exp Surgical Procedures, Operative/ 3453481

4 (surger* or surgical or surgeon* or operat* or preoperativ* or intraoperativ* or perioperativ* or postoperative* or pre-operativ* or intra-operativ* or peri-operativ* or post-operative* or presurg* or intrasurg* or perisurg* or postsurg* or pre-surg* or intra-surg* or peri-surg* or post-surg*).ti,ab,kf. 3274750

5 2 or 3 or 4 5372641

6 Oxygen/ 174285

7 exp Oxygen Inhalation Therapy/ 27957

8 Hyperoxia/ 4201

9 Noninvasive ventilation/ 3376

10 Positive-Pressure Respiration/ 18096

11 Continuous Positive Airway Pressure/ 8754

12 (oxygen* or non invasive ventilation or noninvasive ventilation or non invasive pressure support or noninvasive pressure support or intermittent positive-pressure breathing or intermittent positive pressure breathing or intermittent positive-pressure ventilation or intermittent positive pressure ventilation or nasal cannula* or high flow or highflow or HFNC or HFOC or continuous positive airway pressure or CPAP or continuous positive pressure ventilation or CPPV or bi level positive airway pressure or bilevel positive airway pressure or BiPaP).ti,ab,kf. 669091

13 6 or 7 or 8 or 9 or 10 or 11 or 12 754713

14 5 and 13 108552

15 1 and 14 25394

16 randomized controlled trial.pt. 575518

17 controlled clinical trial.pt. 94999

18 randomized.ab. 573122

19 placebo.ab. 231045

20 clinical trials as topic.sh. 200295

21 randomly.ab. 389788

22 trial.ti. 268920

23 16 or 17 or 18 or 19 or 20 or 21 or 22 1468479

24 exp animals/ not humans.sh. 5039190

25 23 not 24 1350876

26 15 and 25 2978

27 randomized controlled trial.pt. 575518

28 (random* or "controlled trial*" or "clinical trial*" or rct).tw. 1655571

29 27 or 28 1774539

30 exp animals/ not humans.sh. 5039190

31 29 not 30 1625928

32 15 and 31 3121

33 26 or 32 3362

Update – July 2023

Re-ran the search lines above (after changing lines 16 and 27 to exp randomized controlled trial/ to account for the 2023 revision of the Cochrane RCT filters described in the search summary table above)

Search date: 19 July 2023

Applied the following date limit:

34 ("20220823" or "20220824" or "20220825" or "20220826" or "20220827" or "20220828" or "20220829" or "20220830" or "20220831" or 202209* or 202210* or 202211* or 202212* or 2023*).dt,ez,da. [added to database since original search in August 2022] 1644768

35 33 and 34 157

Update – May 2025

Re-ran the search lines above

Search date: 01/05/2025

Applied the following date limit:

34 ("20230719" or "2023072*" or "2023073*" or 202308* or 202309* or 202310* or 202311* or 202312* or 2024* or 2025*).dt,ez,da. [added to database since first update search in July 2023] 3015122

35 33 and 34 344

**Embase (Ovid)**

Note: details of the 2023 and 2025 update searches are at the end of the search string.

Date searched: 23/08/2022

Embase Classic+Embase <1947 to 2022 August 22>

1 exp adolescence/ or exp adolescent/ or exp child/ or exp childhood disease/ or exp infant disease/ or (adolescen* or babies or baby or boy? or boyfriend or boyhood or girlfriend or girlhood or child* or girl? or infan* or juvenil* or juvenile* or kid? or minors or minors* or neonat* or neonat* or neo-nata* or newborn* or new-born* or paediatric* or peadiatric* or pediatric* or perinat* or preschool* or puber* or pubescen* or school or school child* or school* or schoolchild* or schoolchild* or teen* or toddler? or underage? or under-age? or youth*).ti,ab,kw. 6515122

2 exp surgery/ 5927473

3 exp surgeon/ 190614

4 perioperative medicine/ 502

5 (surger* or surgical or surgeon* or operat* or preoperativ* or intraoperativ* or perioperativ* or postoperative* or pre-operativ* or intra-operativ* or peri-operativ* or post-operative* or presurg* or intrasurg* or perisurg* or postsurg* or pre-surg* or intra-surg* or peri-surg* or post-surg*).ti,ab,kw. 4497696

6 2 or 3 or 4 or 5 7571947

7 oxygen/ 269574

8 oxygen therapy/ 41512

9 hyperoxia/ 11037

10 exp noninvasive ventilation/ 20629

11 exp positive pressure ventilation/ 15981

12 (oxygen* or non invasive ventilation or noninvasive ventilation or non invasive pressure support or noninvasive pressure support or intermittent positive-pressure breathing or intermittent positive pressure breathing or intermittent positive-pressure ventilation or intermittent positive pressure ventilation or nasal cannula* or high flow or highflow or HFNC or HFOC or continuous positive airway pressure or CPAP or continuous positive pressure ventilation or CPPV or bi level positive airway pressure or bilevel positive airway pressure or BiPaP).ti,ab,kw. 846646

13 7 or 8 or 9 or 10 or 11 or 12 965568

14 6 and 13 178528

15 Randomized controlled trial/ 725778

16 Controlled clinical study/ 467168

17 Random$.ti,ab. 1836073

18 randomization/ 94934

19 intermethod comparison/ 286523

20 placebo.ti,ab. 350091

21 (compare or compared or comparison).ti. 603153

22 ((evaluated or evaluate or evaluating or assessed or assess) and (compare or compared or comparing or comparison)).ab. 2559665

23 (open adj label).ti,ab. 98998

24 ((double or single or doubly or singly) adj (blind or blinded or blindly)).ti,ab. 265209

25 double blind procedure/ 200358

26 (parallel adj group$1).ti,ab. 29921

27 (crossover or cross over).ti,ab. 119088

28 ((assign$ or match or matched or allocation) adj5 (alternate or group$1 or intervention$1 or patient$1 or subject$1 or participant$1)).ti,ab. 388468

29 (assigned or allocated).ti,ab. 458141

30 (controlled adj7 (study or design or trial)).ti,ab. 419714

31 (volunteer or volunteers).ti,ab. 276693

32 human experiment/ 590473

33 trial.ti. 374846

34 or/16-33 5927416

35 34 not 15 5225021

36 (random$ adj sampl$ adj7 ("cross section$" or questionnaire$1 or survey$ or database$1)).ti,ab. not (comparative study/ or controlled study/ or randomi?ed controlled.ti,ab. or randomly assigned.ti,ab.) 9192

37 Cross-sectional study/ not (randomized controlled trial/ or controlled clinical study/ or controlled study/ or randomi?ed controlled.ti,ab. or control group$1.ti,ab.) 318529

38 (((case adj control$) and random$) not randomi?ed controlled).ti,ab. 20159

39 (Systematic review not (trial or study)).ti. 219285

40 (nonrandom$ not random$).ti,ab. 18119

41 "Random field$".ti,ab. 2766

42 (random cluster adj3 sampl$).ti,ab. 1456

43 (review.ab. and review.pt.) not trial.ti. 1017845

44 "we searched".ab. and (review.ti. or review.pt.) 43249

45 "update review".ab. 120

46 (databases adj4 searched).ab. 52864

47 (rat or rats or mouse or mice or swine or porcine or murine or sheep or lambs or pigs or piglets or rabbit or rabbits or cat or cats or dog or dogs or cattle or bovine or monkey or monkeys or trout or marmoset$1).ti. and animal experiment/ 1162804

48 Animal experiment/ not (human experiment/ or human/) 2443463

49 or/36-48 4033586

50 35 not 49 4569067

51 1 and 14 and 50 5065

Update – July 2023

Re-ran the search lines above (after changing lines 15 to exp Randomized controlled trial/ and removing line 35 to account for the 2023 revision of the Cochrane RCT filters described in the search summary table above)

Search date: 19 July 2023

Applied the following date limit:

51 limit 50 to dc=20220823-20230719 877

Update – May 2025

Re-ran the search lines above (after changing lines 15 to exp Randomized controlled trial/ and removing line 35 to account for the 2023 revision of the Cochrane RCT filters described in the search summary table above)

Actual database (via University of Birmingham) Embase 1974 to 2025 May 01

Search date: 02 May 2025

Applied the following date limit:

51 limit 50 to dc=20230719-20250502 1757

**CENTRAL (Cochrane Library via Wiley)**

Note: details of the 2023 and 2025 update searches are at the end of the search string.

Search Name: Oxygen for surgery paediatrics RCTs

Date Run: 23/08/2022 12:12:42

Comment: 23 August 2022

ID Search Hits

#1 MeSH descriptor: [Specialties, Surgical] explode all trees 2049

#2 MeSH descriptor: [Surgical Procedures, Operative] explode all trees 129429

#3 (preoperativ* or intraoperativ* or perioperativ* or postoperative* or pre-operativ* or intra-operativ* or peri-operativ* or post-operative* or presurg* or intrasurg* or perisurg* or postsurg* or pre-surg* or intra-surg* or peri-surg* or post-surg*):ti,ab,kw 175710

#4 #1 or #2 or #3 244435

#5 MeSH descriptor: [Oxygen] this term only 5477

#6 MeSH descriptor: [Oxygen Inhalation Therapy] explode all trees 1744

#7 MeSH descriptor: [Hyperoxia] this term only 229

#8 MeSH descriptor: [Noninvasive Ventilation] this term only 340

#9 MeSH descriptor: [Positive-Pressure Respiration] this term only 1613

#10 MeSH descriptor: [Continuous Positive Airway Pressure] this term only 1290

#11 (oxygen* or "non invasive ventilation" or "noninvasive ventilation" or "non invasive pressure support" or "noninvasive pressure support" or "intermittent positive-pressure breathing" or "intermittent positive pressure breathing" or "intermittent positive-pressure ventilation" OR "intermittent positive pressure ventilation" or (nasal next cannula*) or "high flow" or highflow or HFNC or HFOC or "continuous positive airway pressure" or CPAP or "continuous positive pressure ventilation" or CPPV or "bi level positive airway pressure" or "bilevel positive airway pressure" or BiPaP):ti,ab,kw 63222

#12 #5 or #6 or #7 or #8 or #9 or #10 or #11 63712

#13 MeSH descriptor: [Adolescent] explode all trees 110478

#14 MeSH descriptor: [Child] explode all trees 61756

#15 MeSH descriptor: [Infant] explode all trees 35072

#16 ((infant next disease*) or (childhood next disease*)):ti,ab,kw 1262

#17 (adolescen* or babies or baby or boy? or boyfriend or boyhood or girlfriend or girlhood or child* or girl? or infan* or juvenil* or kid? or minors or minors* or neonat* or neo-nat* or newborn* or new-born* or paediatric* or peadiatric* or pediatric* or perinat* or preschool* or puber* or pubescen* or school* or teen* or toddler? or underage? or under-age? or youth*) 434163

#18 #4 and #12 and #17 3729

CENTRAL (Trials) 3604

Update – July 2023

Search date: 19 July 2023

Re-ran the search lines above with a date limit

#19 #4 and #12 and #17

with Cochrane Library publication date from Aug 2022 to Jul 2023 235

CENTRAL (Trials) 224

Update – May 2025

Search date: 02/05/2025

Re-ran the search lines above with a date limit

with Cochrane Library publication date from Jul 2023 to May 2025 495

CENTRAL (Trials) 483

# Further details of GRADE assessment

We followed GRADE’s informative statements to communicate results of the review.^1^ Two aspects of a result were used to communicate the review's findings: the size or magnitude of the effect and the level of certainty of the evidence. Two factors are crucially important when determining the size of effect. The first involves calculating and using absolute effects instead of using relative effects that can often be misleading. The second is identifying threshold values in absolute terms which would define the clinical importance of the outcome. We have defined small, moderate and large absolute risk difference to be 1% (10/1000), 2.5% (25/1000) and 5% (50/1000) respectively.

The statements communicate the size of the effect based on the point estimate in a meta-analysis, as it is the most likely value of the true effect given available data. Whether the estimate is sufficiently precise is determined by comparing the confidence intervals against the thresholds for large, moderate and small effects. Wider confidence intervals spanning cross one or more of these thresholds indicate that the estimate may not be sufficiently precise and would result in downgrading of the certainty of evidence. In addition to imprecision, GRADE assessment factors in the risk of bias in the included studies; inconsistency and indirectness of evidence, and the risk of publication bias.

# Figure S1: Effect of high FiO_2_ on PPCs when compared to low FiO_2_


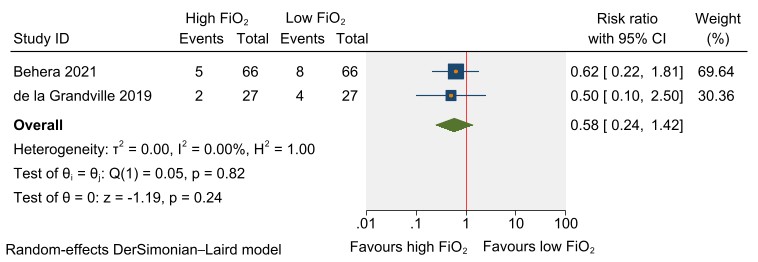


# Figure S2: Effect of high FiO_2_ on PONV when compared to low FiO_2_


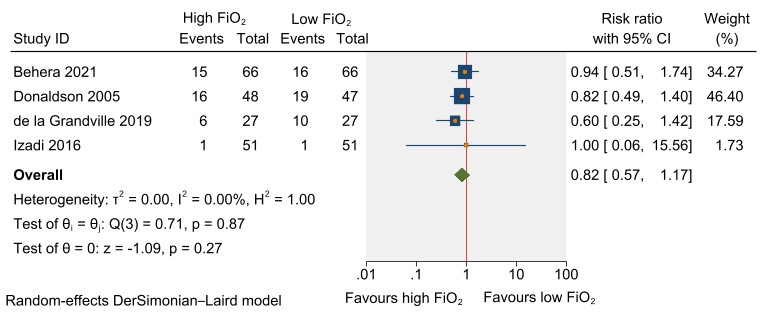


# Figure S3: Effect of HFNO on reintubation when compared to COT


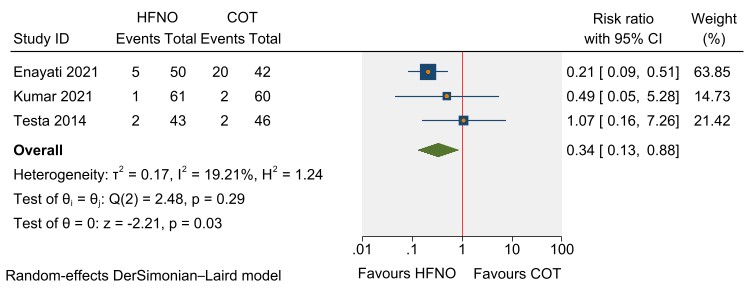


# Figure S4: Effect of HFNO on the incidence of atelectasis when compared to COT


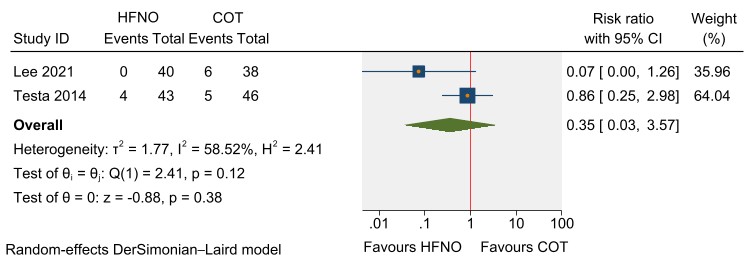


# Figure S5: Effect of HFNO on length of ICU stay when compared to COT


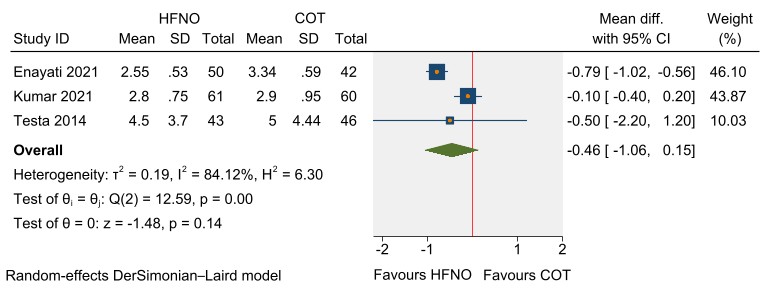


# Figure S6: Effect of HFNO on reintubation when compared to NIV


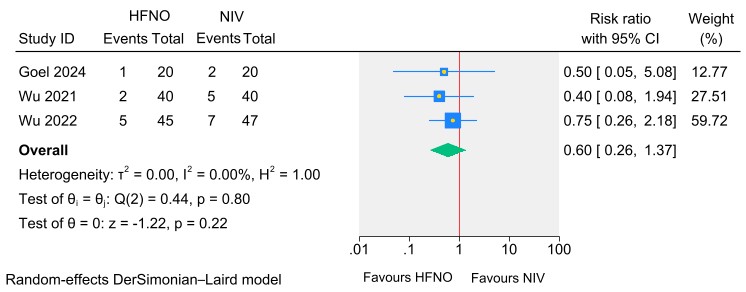


# Figure S7: Effect of HFNO on the incidence of pneumothorax when compared to NIV


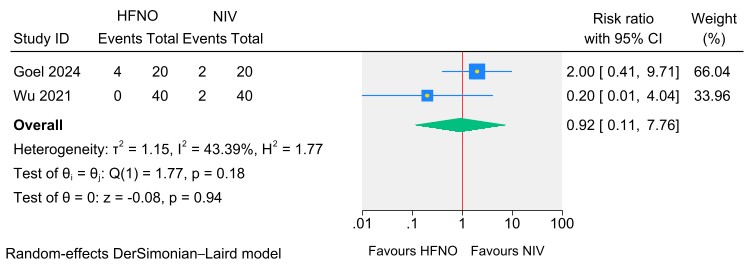


# GRADE evidence profile-High FiO_2_ compared to low FiO_2_

| **Certainty assessment** | | | | | | | **Summary of findings** | | | | |
| --- | --- | --- | --- | --- | --- | --- | --- | --- | --- | --- | --- |
| **Participants (studies) Follow-up** | **Risk of bias** | **Inconsistency** | **Indirectness** | **Imprecision** | **Publication bias** | **Overall certainty of evidence** | **Study event rates (%)** | | **Relative effect (95% CI)** | **Anticipated absolute effects** | |
|  |  |  |  |  |  |  | **With low FiO2** | **With high FiO2** |  | **Risk with low FiO2** | **Risk difference with high FiO2** |
| **Surgical site infection** | | | | | | | | | | | |
| 186 (2 RCTs) | serious^a^ | not serious | not serious | very serious^b^ | none | ⨁◯◯◯ Very low^a,b,c^ | 11/93 (11.8%) | 8/93 (8.6%) | RR 0.75 (0.33 to 1.73) | 11/93 (11.8%) | **30 fewer per 1,000** (from 79 fewer to 86 more) |
| **Postoperative pulmonary complications** | | | | | | | | | | | |
| 186 (2 RCTs) | serious^a^ | not serious | not serious | very serious^b^ | none | ⨁◯◯◯ Very low^a,b,c^ | 12/93 (12.9%) | 7/93 (7.5%) | RR 0.58 (0.24 to 1.42) | 12/93 (12.9%) | **54 fewer per 1,000** (from 98 fewer to 54 more) |
| **Postoperative nausea and vomiting** | | | | | | | | | | | |
| 383 (4 RCTs) | serious^a^ | not serious | not serious | very serious^b^ | none | ⨁◯◯◯ Very low^a,b,c^ | 46/191 (24.1%) | 38/192 (19.8%) | RR 0.82 (0.57 to 1.17) | 46/191 (24.1%) | **43 fewer per 1,000** (from 104 fewer to 41 more) |
| **Atelectasis** | | | | | | | | | | | |
| 86 (1 RCT) | serious^c^ | not serious | not serious | extremely serious^d^ | none | ⨁◯◯◯ Very low^d,e^ | 12/43 (27.9%) | 16/43 (37.2%) | RR 1.33 (0.72 to 2.47) | 12/43 (27.9%) | **92 more per 1,000** (from 78 fewer to 410 more) |

**CI:** confidence interval; **RR:** risk ratio

#### Explanations

a. Studies were at high/unclear risk of bias

b. Wide confidence interval suggesting appreciable benefit and appreciable harm.

c. The study was deemed as having some concerns for risk of bias

d. Single study with very wide confidence interval

# GRADE evidence profile- HFNO compared to COT

| **Certainty assessment** | | | | | | | **Summary of findings** | | | | |
| --- | --- | --- | --- | --- | --- | --- | --- | --- | --- | --- | --- |
| **Participants (studies) Follow-up** | **Risk of bias** | **Inconsistency** | **Indirectness** | **Imprecision** | **Publication bias** | **Overall certainty of evidence** | **Study event rates (%)** | | **Relative effect (95% CI)** | **Anticipated absolute effects** | |
|  |  |  |  |  |  |  | **With COT** | **With HFNO** |  | **Risk with COT** | **Risk difference with HFNO** |
| **Reintubation** | | | | | | | | | | | |
| 302 (3 RCTs) | serious^a^ | not serious | not serious | serious^b^ | none | ⨁⨁◯◯ Low | 24/148 (16.2%) | 8/154 (5.2%) | RR 0.34 (0.13 to 0.88) | 162 per 1,000 | **107 fewer per 1,000** (from 141 fewer to 19 fewer) |
| **Atelectasis** | | | | | | | | | | | |
| 167 (2 RCTs) | serious^a^ | serious^c^ | not serious | very serious^b^ | none | ⨁◯◯◯ Very low | 11/84 (13.1%) | 4/83 (4.8%) | RR 0.35 (0.03 to 3.57) | 131 per 1,000 | **85 fewer per 1,000** (from 127 fewer to 337 more) |
| **Length of ICU stay** | | | | | | | | | | | |
| 302 (3 RCTs) | serious^a^ | serious^c^ | not serious | serious^b^ | none | ⨁◯◯◯ Very low | 148 | 154 | - |  | MD 0.46 lower (1.06 lower to 0.15 higher) |

**CI:** confidence interval; **MD:** mean difference; **RR:** risk ratio

#### Explanations

a. Studies were at high/unclear risk of bias

b. Wide confidence interval suggesting appreciable benefit and appreciable harm.

c. Significant heterogeneity was observed

# GRADE evidence profile- NIV compared to COT

| **Certainty assessment** | | | | | | | **Summary of findings** | | | | |
| --- | --- | --- | --- | --- | --- | --- | --- | --- | --- | --- | --- |
| **Participants (studies) Follow-up** | **Risk of bias** | **Inconsistency** | **Indirectness** | **Imprecision** | **Publication bias** | **Overall certainty of evidence** | **Study event rates (%)** | | **Relative effect (95% CI)** | **Anticipated absolute effects** | |
|  |  |  |  |  |  |  | **With COT** | **With NIV** |  | **Risk with COT** | **Risk difference with NIV** |
| **Unplanned ICU admission** | | | | | | | | | | | |
| 81 (1 RCT) | serious^a^ | not serious | not serious | extremely serious^b^ | none | ⨁◯◯◯ Very low^a,b^ | 12/41 (29.3%) | 10/40 (25.0%) | RR 0.85 (0.42 to 1.75) | 12/41 (29.3%) | **44 fewer per 1,000** (from 170 fewer to 220 more) |
| **Intubation rate** | | | | | | | | | | | |
| 81 (1 RCT) | serious^a^ | not serious | not serious | extremely serious^b^ | none | ⨁◯◯◯ Very low^a,b^ | 7/41 (17.1%) | 10/40 (25.0%) | RR 1.46 (0.62 to 3.47) | 7/41 (17.1%) | **79 more per 1,000** (from 65 fewer to 422 more) |
| **Atelectasis** | | | | | | | | | | | |
| 41 (1 RCT) | serious^a^ | not serious | not serious | extremely serious^b^ | none | ⨁◯◯◯ Very low^a,b^ | 6/20 (30.0%) | 2/21 (9.5%) | RR 0.32 (0.07 to 1.39) | 6/20 (30.0%) | **204 fewer per 1,000** (from 279 fewer to 117 more) |
| **Postoperative vomiting** | | | | | | | | | | | |
| 40 (1 RCT) | serious^a^ | not serious | not serious | extremely serious^b^ | none | ⨁◯◯◯ Very low^a,b^ | 11/20 (55.0%) | 9/20 (45.0%) | RR 0.82 (0.44 to 1.53) | 11/20 (55.0%) | **99 fewer per 1,000** (from 308 fewer to 292 more) |

**CI:** confidence interval; **RR:** risk ratio

#### Explanations

a. The study was deemed as having some concerns for risk of bias

b. Very wide confidence interval suggesting appreciable benefit and appreciable harm

# GRADE evidence profile- HFNO compared to NIV

| **Certainty assessment** | | | | | | | **Summary of findings** | | | | |
| --- | --- | --- | --- | --- | --- | --- | --- | --- | --- | --- | --- |
| **Participants (studies) Follow-up** | **Risk of bias** | **Inconsistency** | **Indirectness** | **Imprecision** | **Publication bias** | **Overall certainty of evidence** | **Study event rates (%)** | | **Relative effect (95% CI)** | **Anticipated absolute effects** | |
|  |  |  |  |  |  |  | **With NIV** | **With HFNO** |  | **Risk with NIV** | **Risk difference with HFNO** |
| **Reintubation** | | | | | | | | | | | |
| 212 (3 RCTs) | serious^a^ | not serious | not serious | very serious^b^ | none | ⨁◯◯◯ Very low^a,b^ | 14/107 (13.1%) | 8/105 (7.6%) | RR 0.60 (0.26 to 1.37) | 14/107 (13.1%) | **52 fewer per 1,000** (from 97 fewer to 48 more) |
| **Pneumothorax** | | | | | | | | | | | |
| 120 (2 RCTs) | serious^a^ | not serious | not serious | extremely serious^b^ | none | ⨁◯◯◯ Very low^a,b^ | 4/60 (6.7%) | 4/60 (6.7%) | RR 0.92 (0.11 to 7.76) | 4/60 (6.7%) | **5 fewer per 1,000** (from 59 fewer to 451 more) |
| **Respiratory failure** | | | | | | | | | | | |
| 80 (1 RCT) | serious^c^ | not serious | not serious | extremely serious^b^ | none | ⨁◯◯◯ Very low^b,c^ | 9/40 (22.5%) | 3/40 (7.5%) | RR 0.33 (0.10 to 1.14) | 9/40 (22.5%) | **151 fewer per 1,000** (from 203 fewer to 31 more) |
| **Hospital mortality** | | | | | | | | | | | |
| 80 (1 RCT) | serious^c^ | not serious | not serious | extremely serious^b^ | none | ⨁◯◯◯ Very low^b,c^ | 1/40 (2.5%) | 0/40 (0.0%) | RR 0.33 (0.01 to 7.95) | 1/40 (2.5%) | **17 fewer per 1,000** (from 25 fewer to 174 more) |

**CI:** confidence interval; **RR:** risk ratio

#### Explanations

a. Studies were at high/unclear risk of bias

b. Wide confidence interval suggesting appreciable benefit and appreciable harm.

c. The study was deemed as having some concerns for risk of bias

1. Santesso N, Glenton C, Dahm P, et al. GRADE guidelines 26: informative statements to communicate the findings of systematic reviews of interventions. *Journal of clinical epidemiology* 2020;119:126-35.
